# Viscoelastic properties and efficient acoustic damping in confined polymer nano-layers at GHz frequencies

**DOI:** 10.1038/srep33471

**Published:** 2016-09-16

**Authors:** Mike Hettich, Karl Jacob, Oliver Ristow, Martin Schubert, Axel Bruchhausen, Vitalyi Gusev, Thomas Dekorsy

**Affiliations:** 1 Department of Physics and Center for Applied Photonics, University of Konstanz, Germany; 2 Instituto Balseiro & Centro Atomico Bariloche (CNEA), and CONICET, Argentina; 3 LUNAM Universités, CNRS, Université du Maine, LAUM UMR-CNRS 6613, Av.O. Messiaen, 72085 Le Mans, France; 4 Institute of Technical Physics, German Aerospace Center, Pfaffenwaldring 38-40, 70568 Stuttgart, Germany

## Abstract

We investigate the viscoelastic properties of confined molecular nano-layers by time resolved optical pump-probe measurements. Access to the elastic properties is provided by the damping time of acoustic eigenmodes of thin metal films deposited on the molecular nano-layers which show a strong dependence on the molecular layer thickness and on the acoustic eigen-mode frequencies. An analytical model including the viscoelastic properties of the molecular layer allows us to obtain the longitudinal sound velocity as well as the acoustic absorption coefficient of the layer. Our experiments and theoretical analysis indicate for the first time that the molecular nano-layers are much more viscous than elastic in the investigated frequency range from 50 to 120 GHz and thus show pronounced acoustic absorption. The longitudinal acoustic wavenumber has nearly equal real and imaginary parts, both increasing proportional to the square root of the frequency. Thus, both acoustic velocity and acoustic absorption are proportional to the square root of frequency and the propagation of compressional/dilatational acoustic waves in the investigated nano-layers is of the diffusional type, similar to the propagation of shear waves in viscous liquids and thermal waves in solids.

The viscoelastic behavior of ultrathin polymers especially under 1-d confinement plays an important role in their glass transition dynamics^1^. Due to strong interface contributions and confinement effects, capped polymer layers (interfaces at both sides) and supported ones (deposited onto a solid substrate) exhibit considerable deviations from the bulk behaviour^2^,^3^,^4^,^5^. Yet, the structural dynamics of ultrathin polymer films still pose many open questions^1^. In addition, a better understanding of the mechanical and thermal transport properties of these films is crucial for advances in nanotechnology especially for phonon engineering.

The transport and damping of heat and acoustic waves across metal-molecule interfaces is also important for applications of thin film and nanoparticle-polymer/molecule hybrid systems in fundamental research, nanotechnology, and medicine, for example in photoacoustic imaging and cancer treatment. Here, molecules are often used in combination with metallic nanoparticles to mark and address specific cell types^6^. Furthermore, the influence of surface adhesion^7^, interface stiffness between nanoparticles^8^, and of the phononic properties of the material constituents^9^ have been shown to be of considerable importance for heat and acoustic phonon transport. All of these properties are also intimately related to the mechanical and thus viscoelastic properties of ultrathin polymer layers and can therefore be utilized in the design of acoustic and thermal nanodevices.

In this work we study the viscoelastic properties of ultrathin polymer layers by investigation of their influence on the coherent acoustic phonon transport in stratified systems. This also allows us to obtain information about the acoustic sound velocity and the damping of acoustic waves inside the polymer layer which is of special interest for phonon engineering. The molecular layers are confined between a gold film and a silicon substrate which alters the acoustic interface resistance. When the gold film is excited by an ultrashort laser pulse it starts coherent oscillations with a damping time determined by a pump-probe experiment. The observed change in the acoustic damping time with the molecular layer thickness is modelled analytically including the viscoelastic properties of the molecular layer.

The mechanical properties at GHz frequencies of these layers are difficult to address with other methods, especially in the confined geometry with thin (<15 nm) layers studied in this work. We choose aminopropyltrichlorosilane (APTES) as the organic interface layer due to its wide use in nanotechnology as adhesion promoter^10^,^11^. This type of molecule allows to grow films down to monolayer thickness via self-assembly and the amino group of the molecule hinders gold diffusion into the molecular layer.

The structure of the sample is shown in Fig. 1(a). Molecule layers with varying thicknesses are confined between a silicon substrate and a gold capping layer. A native SiO_2_ layer is present at the silicon surface.

In order to investigate the coherent acoustic phonon dynamics in these systems we employ time resolved optical pump-probe spectroscopy. An optical pulse excites the coherent dynamics in the sample, which are subsequently probed by measuring the reflectivity of a second, weaker and time delayed optical pulse. This yields the time resolved optical response of the sample including the temporal evolution of the excited coherent longitudinal (compression/dilatation) phonons. Details of the experimental setup are given in the Methods section.

A typical result of a pump-probe experiment is shown in Fig. 1(b). At zero time delay the pump- and probe pulse coincide on the sample. The immediate sharp drop of the signal is caused by the ultrafast heating of the electrons and the accompanying change in the optical properties of the gold film. The electrons thermalize via electron-electron and electron-phonon interaction where the latter causes an impulsive heating of the gold film and thus excites a coherent vibrational mode in the gold film by the thermoelastic process.

The acoustic mode of the system gives rise to a periodic modulation of the transient reflectivity due to a change in optical properties caused by the strain in the sample and the resulting photoelastic response. These oscillations are well visible in the inset in Fig. 1(b). In order to obtain the damping time of the oscillation we first remove the electronic background and subsequently perform a least squares fit with the function:


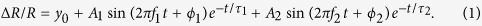


The first oscillatory part describes the damped coherent thickness oscillation of the gold film with the frequency *f*_1_ ≈ *v*_Au_/(2*D*), where the longitudinal sound velocity and the gold film thickness are denoted by *v*_Au_ and *D*, respectively. The second oscillatory component, usually barely visible, stems from the time resolved Brillouin scattering in the silicon substrate caused by the interference between light reflected of the sample interfaces and the propagating acoustic pulses. Finally, *y*_0_ accounts for a static offset. The extracted mode with the superimposed fit is presented in Fig. 1(c). As will be discussed later on, the mode frequency *f*_1_ is only slightly influenced by the molecular layer.

## Results and Discussion

We measured the extracted damping time *τ*_1_ (see eqn. 1) of the fundamental gold film thickness mode for molecular layer thicknesses ranging from 1.8 to 13.8 nm, i.e., spanning nearly one order in thickness variation. The expected thickness^12^, i.e., given by the length of the molecule, would be 0.7 nm for an ideal APTES monolayer. The discrepancy to the thinnest layers obtained experimentally (1.8 nm) is attributed to the uncertainty in the APTES layer thickness measurement which is discussed in detail in the Methods section. The investigated frequency range, given by the thickness of the gold film, covers 50 to around 120 GHz.

**Figure 2:**
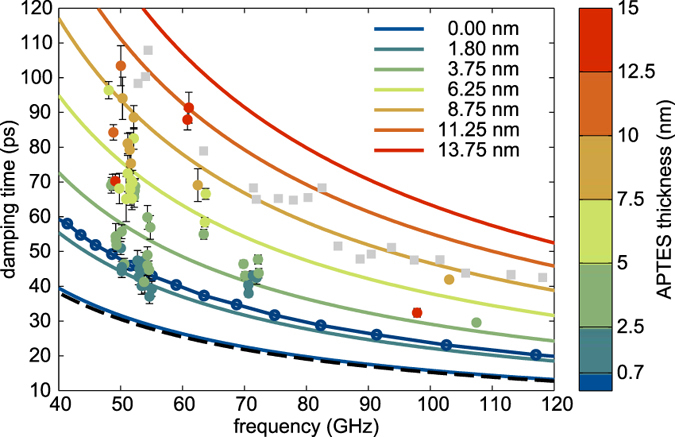
Acoustic mode damping times as a function of mode frequency. The respective APTES layer thickness is indicated by the color coding. Solid lines present the results of the viscoelastic modelling. Grey squares depict reproduced results from Hettich *et al*.^13^ for comparison. The black dashed line shows the analytic results for a single gold layer on silicon and the dark blue line with circles depicts the calculated damping times for an Au/SiO_2_/Si layer system.

The extracted damping times are presented in Fig. 2 as a function of the mode frequency and the respective APTES layer thickness is indicated by the color-coding. Also shown are the theoretical results as full and dashed lines which will be discussed later on. Immediately visible is a striking dependence of the obtained damping times on the thickness of the molecular layer, which acts as a barrier for the coherent phonons on their way from the gold film generator to the substrate.

For thicker molecular layers an increase in the mode damping time by almost a factor of three is observed at 50 GHz. The frequency dependence shows a similar behaviour as previously published results^13^. These results are plotted as grey squares for comparison. We also observe that the damping times approach the expected damping times of the Au/SiO_2_/Si layer system shown as solid line with circles, when the thickness of the molecular nano-layer is approaching zero. Despite beeing usually neglected, this suggests to include the native silicon oxide layer in future extensions of the here presented model.

**Figure 3:**
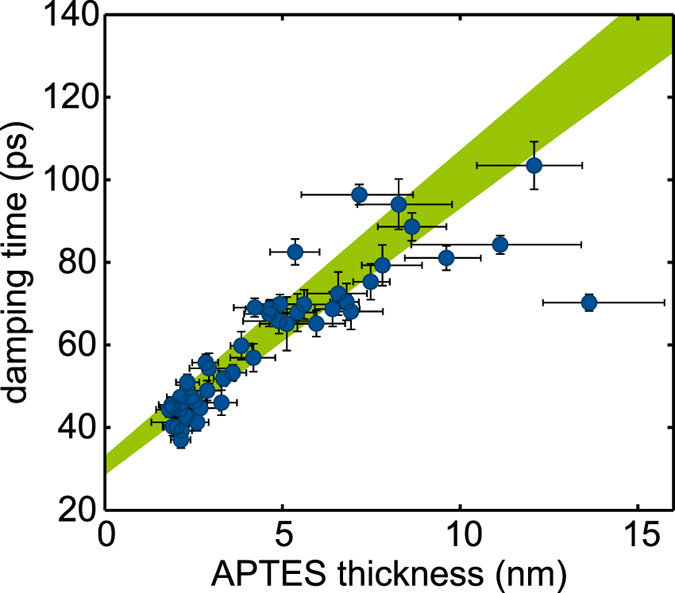
Damping time of thickness mode as a function of the APTES thickness. The data range from 48–55 GHz, the explicit frequency dependence is omitted here for better visibility. The green cone shows the results of the viscoelastic simulation also in the range between 48–55 GHz.

The data around 50 GHz are shown in a different representation in Fig. 3. Here, the damping time is plotted versus the APTES thickness. The frequency dependence is omitted here for clarity and the green filled cone shows the theoretical results discussed later on. The damping times follow a nearly linear trend until APTES layer thicknesses of 8–9 nm. For thicker APTES layers the damping times seem to saturate. Unfortunately, our preparation method does not allow for thicker APTES layer thicknesses to investigate this regime further.

The strong modification of the acoustic mode damping time allows us to access the viscoelastic properties of the molecular layer by calculation of the mechanic eigenmodes of the layered system and their respective damping times. The model takes into account a two layer system attached to a semi-infinite substrate and the viscoelastic properties are included by means of the standard viscoelastic solid model^14^ for the polymer layer. This model includes three parameters that describe the viscoelastic behaviour of the molecular layer: the elastic modulus *L*_0_ for low frequencies, the elastic modulus *L*_∞_ for infinitely high frequencies, and the intrinsic relaxation time *τ*. We obtain a readily usable analytic expression assuming that the gold mode frequency is only slightly influenced by the molecular layer. This assumption is corroborated by measurements on patterned APTES layer systems^15^ where we observed negligible frequency shifts with or without APTES layer between the gold film and the substrate. The details regarding the theoretical calculations are given in the Theory section.

We use the APTES thickness dependence of the data centered around 50 GHz (Fig. 3) to find the best agreement between our model and the experimental data. Due to a strong correlation between *L*_0_ and *τ* we choose *L*_0_ to be in reasonable agreement with results reported in literature on solution deposited APTES layers where layers down to 110 nm thickness were studied^16^. The found viscoelastic parameters are given in Table 1 and those used in the calculations are given in Table 2. A good agreement between the theoretical results shown in Fig. 2 (solid lines) and Fig. 3 (green cone) as well as for the gold mode frequency dependence and for the APTES thickness dependence is achieved.

An important finding is the fact that the simulation does not require an explicit thickness dependence of the three parameters, *i.e.*, one parameter set is sufficient to reproduce the experimental results in the range from 1.8 and around 9 nm. There are too few measurements for thicker layers where the damping times seem to saturate in order to include them into the data fitting. The extension to thicker layers is an important task for future work.

**Figure 4:**
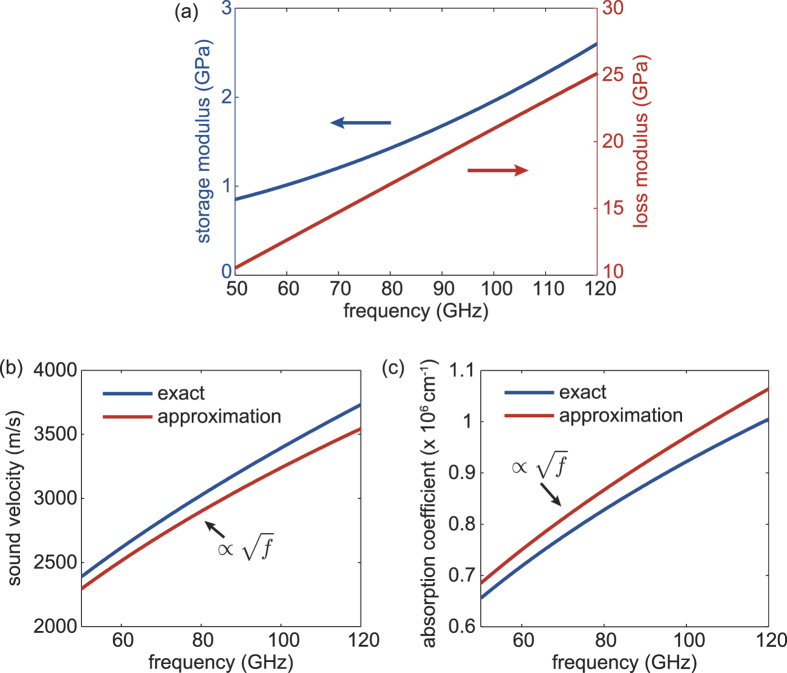
Frequency dependence of (**a**) the storage modulus and loss modulus, (**b**) the sound velocity in the APTES layer, and (**c**) the acoustic absorption coefficient in the APTES layer.

We also show the frequency dependence of the storage *L*′ and the loss modulus *L*′′ of the polymer layer in the investigated frequency range obtained by the viscoelastic model in Fig. 4(a). This allows us to calculate (see Theory section) the frequency dependent sound velocity *v*_APTES_ and the acoustic absorption coefficient *α* in the polymer layer. These are shown in Fig. 4(b,c), respectively. The blue lines show the results obtained by the calculation performed with the data in (a) while the red lines show the results of an approximation to the used model that emphasises the underlying physics and will be discussed in the following.

Our approach yields acoustic sound velocities at 50 GHz which are similar to those found in the works of Morath *et al*.^17^ and Akimov *et al*.^18^. However, we find an increased acoustic absorption coefficient in the polymer layer compared to their observations. Taking into account that the polymer layer shows a mainly viscous behaviour, i.e., *L*′/*L*′′ ≈ 0.1, the frequency dependent sound velocity as well as the absorption coefficient are in a good approximation proportional to *f*^1/2^ (see Theory section) as depicted in (b) and (c). The offset in absolute values is on the order of 5% as expected by the *L*′/*L*′′ ratio but the frequency dependence exhibits an excellent agreement with our findings. The real and imaginary parts of the acoustic wavenumber are nearly equal.

This is an interesting result as it implicates that the observed acoustic propagation in the investigated nano-layers is characteristic to diffusion-type waves, such as transverse acoustic waves in viscous liquids or thermal waves in solids^19^. This finding is in contrast to results obtained on much thicker polymer films in a similar frequency range where a linear^17^,^18^ or square dependence^17^ is observed.

In addition, this finding also gives a possible explanation for the saturation effect observed for the damping times of thicker polymer layers. The estimated penetration depth *λ* = 1/*α* of the acoustic wave in the polymer layer is around 15 nm which is close to our experimental observation. Thus, if the polymer layer thickness exceeds 15 nm the acoustic wave is not influenced any more by the substrate because it is attenuated before reaching the substrate. Our data shows that this regime begins around 9 nm. Thus, strong attenuation of the diffusion-type acoustic waves in the molecular nano-layers studied here can provide complete isolation of the films from the substrates by molecular layers of 15 nm thickness.

It is tentative to attribute the mentioned differences in the sound absorption laws to the structural difference of the self-assembled APTES layers, studied by us, from the completely disordered glasses and polymers studied by Morat^17^ and Akimov^18^. However, the nanoscale origin/mechanism of the revealed acoustic absorption definitely requires deep theoretical study which is beyond the scope of the current report.

## Theory

**Figure 5:**
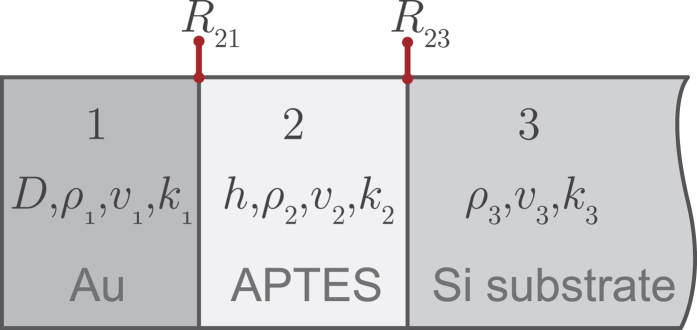
Schematic of the layer system and respective definitions for the theoretical modelling.

We provide in this section the theoretical framework that allows to model the frequency as well as the APTES thickness dependence of the fundamental gold film thickness mode. A mechanic continuum theory, where the viscoelastic properties of the molecule layer are taken into account, provides the acoustic eigenmodes of the three layer system with their respective damping times. The use of continuum mechanic model is justified by experimental results corroborating the validity of such models down to few nanometer dimension of nano-objects^20^. We include the viscoelastic properties by the standard model^14^ and neglect in this description the native SiO_2_-layer covering the silicon substrate. The sample geometry with the used definitions is shown in Fig. 5 where the thicknesses of layer 1 and layer 2 are denoted by *D* and *h* respectively. The layer densities are given by *ρ*_*i*_, the sound velocities by *v*_*i*_ and the wave numbers by *k*_*i*_ = *ω*/*v*_*i*_ where *ω* = *ω*′ + i*ω*′′ are the complex eigenfrequencies of the layer system. The real part *ω*′ describes the eigenmode frequencies while the imaginary part *ω*′′ accounts for the damping of the mode.

The acoustic eigenmodes and the respective damping time of the three layer system are then given by


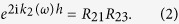


The coefficient *R*_23_ is the reflection coefficient for the acoustic wave incident from the molecular nano-layer, (2), on the semi-infinite substrate, (3), while the coefficient *R*_21_ is the reflection coefficient for the acoustic wave incident from the nano-layer on the film, (1), of finite thickness, *i.e.*, it accounts for the waves travelling inside the film and returning into the molecular nano-layer. Note, that the reflection coefficients introduced in Equation 2 are defined for the mechanical displacement in the acoustic wave. Assuming perfect interface bonding, which is a reasonable assumption due to the strong interface coupling by the molecules, these are given by


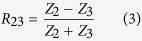


and


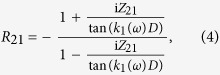


with the acoustic impedances *Z*_*i*_ = *ρ*_*i*_*v*_*i*_ of the respective layers and *Z*_*ij*_ = *Z*_*i*_/*Z*_*j*_. The eigenfrequencies of the layer system and respective damping times can only be obtained numerically by solving


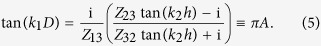


However, an analytic solution for the special case when the eigenfrequency of the layer system is only weakly influenced by the molecular layer is presented in the following. For this special case the complex frequency can be written as


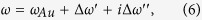


where Δ*ω*′ is a small deviation from the layer eigenfrequencies *ω*_*Au*_ of a single layer system. The eigenfrequency shift Δ*ω*′ and damping rate Δ*ω*′′ can then be calculated from:


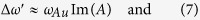



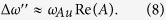


In order to account for viscoelastic properties of the molecular layer a complex elastic modulus *L* = *L*′ + i*L*′′ is introduced for layer 2. Thus the acoustic impedance can be written as


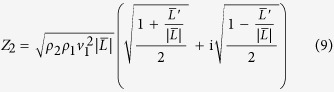


and the product *k*_2_*h* is calculated by


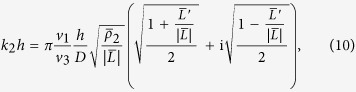


where the barred variables are given by


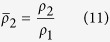



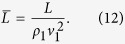


The standard model^14^ for the viscoelastic solid yields for the real and imaginary part of the longitudinal modulus *L*:


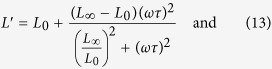



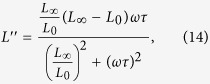


where *L*′ is referred to as *storage modulus* and *L*′′ as *loss modulus*. The moduli *L*_0_ and *L*_∞_ describe the elastic modulus of the system for slowly and fast varying stress respectively, while *τ* describes the characteristic relaxation time of the system. Thus the model has three free parameters *L*_0_, *L*_∞_ and *τ* that yield the viscoeleastic properties of the molecular layer. The model yields results close to the analytic expression when no confined layer is present, *i.e.*, just a gold film on a silicon substrate. This is evident by the dashed line in Fig. 2, which shows the analytic results and is almost identical to the theoretical results we obtain for zero APTES layer thickness.

In the following we present some approximations and estimates to the discussed theory which result from the obtained viscoelastic parameters.

### Estimates

The analyzed data yields *L*_∞_ ≈ 300 GPa and *L*_0_ ≈ 0.5 GPa, thus the ratio *L*_∞_/*L*_0_ ≈ 600 and 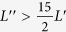 in the investigated frequency range allows us to estimate


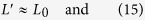



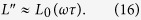


Consequently, we obtain for the sound velocity *v*_2_ = *v*_APTES_ in the polymer layer


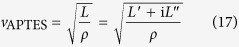



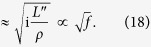


From this follows for the wave number *k*_2_ and the acoustic absorption coefficient *α*


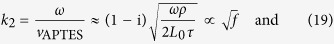



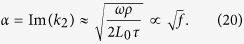


It is worth pointing out that we obtain a *f* ^1/2^ proportionality of the sound velocity and the acoustic absorption coefficient in the APTES layer, which indicates a diffusion-type behavior of the compressional/dilatational acoustic waves in the studied molecular nano-layers.

## Conclusions

In conclusion, we have investigated the viscoelastic properties of ultrathin confined polymer layers (APTES) by coherent acoustic phonon spectroscopy. Thin polymer films in the range between 1.8 to 13.8 nm, i.e., for a thickness varying nearly one order of magnitude, are studied at GHz frequencies. We observe no distinct thickness dependence of the viscoelastic properties of the layer below 9 nm but observe first indications of a change in these properties for thicker layers. The obtained frequency dependent sound velocity between 2390–3730 m/s is in reasonable agreement with earlier measurements in thicker layers. However, we find a higher acoustic absorption coefficient in the polymer that follows a *f*^1/2^ dependency. This is in contrast to reported findings of linear^17^,^18^ or quadratic^17^ dependencies. Our results indicate that the propagation of longitudinal acoustic waves at GHz frequencies in our molecular nano-layers resembles those of diffusion-type waves exhibiting nearly equal real and imaginary parts of the wavenumber. The strong acoustic absorption and the newly observed acoustic properties of the investigated ultrathin polymer layers considerably expand the possibilities of their use in the acoustic and thermal design of nanodevices.

## Methods

### Sample Preparation and Characterization

#### Sample Preparation

All molecular layers are prepared on (100) oriented silicon wafers with a thickness of 500 μm. The wafer is cut into pieces of 0.5 mm × 1 mm size. In order to protect the surface, the wafer is coated with a PMMA layer prior to the cutting. Several cleaning steps are conducted before the molecules are assembled. First, sonication in acetone is used to remove the protective PMMA layer and dirt particles. Subsequently the RCA (Radio Corporation of America)^21^ cleaning method is applied to remove organic and anorganic debris. The cleaning steps are listed below in more detail:

1. 30 min ultrasonication in acetone
2. 10 min RCA cleaning in a solution of 5 H_2_O:1 H_2_O_2_:1 NH_4_OH at 150 °C
3. 10 min sonication in ultrapure water
4. 30 min in a solution of 6 H_2_O:1 H_2_O_2_:1 HCL at 150 °C
5. 10 min sonication in ultrapure water.

After these steps the samples are dried with argon gas and are then transferred into an oxygen plasma cleaner to remove possible organic residues. The samples are then treated with argon gas again and are transferred into a chamber with nitrogen atmosphere where they are immersed in the molecule solution consisting of 12 μL APTES (purchased from *Sigma Aldrich*) and 50 mL toluene. The immersion time can be varied to allow for a rough APTES thickness adjustment and ranges here from 1 h to 160 h. Rinsing with pure toluene and a further sonication process lasting 5 in chloroform after removing the sample from the nitrogen atmosphere eradicates residual non-bonded molecules. As a final step, a snowjet method^22^ is used to get rid of possible residual molecule conglomerates from the surface.

#### Characterization and Selection of Measurement Positions

The index of refraction for APTES is very similar to that of native SiO_2_. Therefore, we measured the native SiO_2_ thickness on test samples which are cleaned in the same batch and use this value for the determination of the APTES layer thickness by ellipsometry. AFM measurements reveal that our molecular layers exhibit uniform thicknesses on a 2 micron scale with thickness variations on larger scales. This allows us to measure several molecular layer thicknesses on the same samples which is a major advantage as this rules out differences in sample preparation (which is known to be very sensitive) as origin of the observed effects. Additional friction force measurements show an overall coverage of the silicon wafer by the APTES layer in the limits of our resolution.

Special care was taken to identify molecular layer regions with uniform thicknesses by ellipsometry. Each sample was characterized at 36 positions. At each of these locations 5 measurements were taken and the areas which showed the most homogeneous thickness distribution in the respective area were chosen for further measurements.

### Asynchronous Optical sampling

Our method of choice to conduct femtosecond time-resolved experiments is asynchronous optical sampling (ASOPS)^23^. This is a modified pump-probe method where the time delay between the pump and the probe pulse is realized by a locked frequency offset Δ*f* = 5 kHz between the two used 800 MHz Ti:sapphire oscillators. Time resolutions of sub-50 fs have been demonstrated for this kind of system^24^. The results reported here are however limited to about 300 fs time resolution due to optical pulse dispersion in some of the optical components in our setup. The optical spotsizes have a FWHM of about 2 micron which allows us to selectively address regions of homogeneous molecular layer thickness on the samples. The experiments are conducted with pump and probe wavelengths of 790 nm and 820 nm, respectively, and allows to suppress residual pump light by color filtering.

## Additional Information

**How to cite this article**: Hettich, M. *et al*. Viscoelastic properties and efficient acoustic damping in confined polymer nano-layers at GHz frequencies. *Sci. Rep.* **6**, 33471; doi: 10.1038/srep33471 (2016).

## Figures and Tables

**Figure 1:**
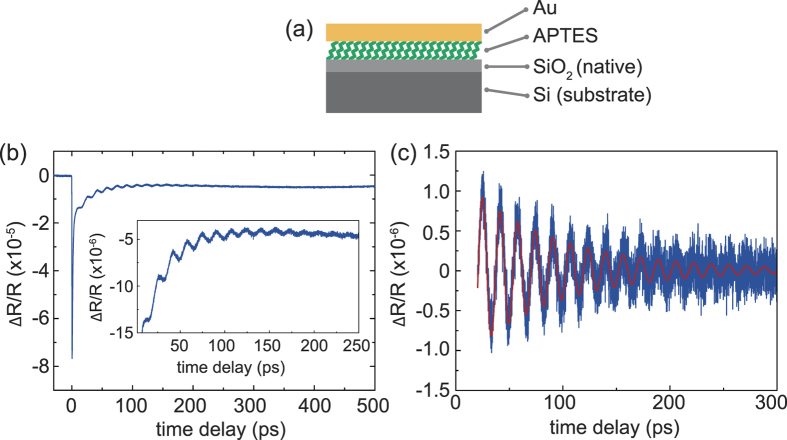
(**a**) Sample sketch (**b**) time domain signal showing a sharp drop at zero time delay due to heated electrons and the subsequent relaxation dynamics including the periodic modulation of the signal due to coherent acoustic phonons (**c**) coherent acoustic phonons after background removal with superimposed fit (eqn. 1) as solid red line.

**Table 1:** Parameters of the viscoelastic simulation for the APTES layer.

*L*_0_ (GPa)	*L*_∞_ (GPa)	*τ* (ps)
0.48	300	70

**Table 2:** Sound velocities and densities used for the viscoelastic simulation.

Material	Sound velocity *v*_*l*_ (ms^−1^)	Density *ρ* (kg m^−3^)
Gold	3240^25^	19200^25^
Silicon	8430^17^	2340^17^
SiO_2_	5850^26^	2170^26^
APTES	—	1000

The frequency dependent sound velocity of the APTES layer is given by the viscoelastic simulation (Fig. 4(b)).
